# Forensic analytical aspects of homemade explosives containing grocery powders and hydrogen peroxide

**DOI:** 10.1038/s41598-024-51335-w

**Published:** 2024-01-07

**Authors:** Tomasz Otłowski, Maciej Zalas, Błażej Gierczyk

**Affiliations:** Faculty of Chemistry, Adam Mickiewicz University, Poznań, 8 Uniwersytetu Poznańskiego Str., 61-614 Poznań, Poland

**Keywords:** Analytical chemistry, Chemical safety, Energy

## Abstract

Homemade explosives become a significant challenge for forensic scientists and investigators. In addition to well-known materials such as acetone peroxide trimer, black powder, or lead azides, perpetrators often produce more exotic and less recognized Homemade Explosives (HMEs). Mixtures of hydrogen peroxide with liquid fuels are widely acknowledged as powerful explosives. Interestingly, similar explosive properties are found in mixtures of numerous solid materials with H_2_O_2_. Notably, powdered groceries, such as coffee, tea, grounded spices, and flour, are particularly interesting to pyrotechnics enthusiasts due to their easy production using accessible precursors, which do not attract the attention of security agencies. H_2_O_2_-based HMEs may become a dangerous component of improvised explosive devices for terrorists and ordinary offenders. For the four most powerful mixtures—HMEs based on coffee, tea, paprika, and turmeric—molecular markers useful for identification using the GC–MS technique have been proposed. Furthermore, the observed time-dependent changes in mixtures of H_2_O_2_ with these food products were studied and evaluated as a potential method for assessing the age of the evidence and reconstructing timelines of crimes. The paper also discusses the usefulness of FT-IR spectroscopy for identifying H_2_O_2_-based HMEs.

## Introduction

Homemade explosives (HMEs) and improvised explosive devices (IEDs) pose an increasing challenge for public safety agencies, capturing the interest of various individuals and groups^1–16^. HMEs are manufactured or assembled for a multitude of reasons, including organized crime, terrorism, actions by mentally ill individuals, and acts of vengeance. Non-professional pyrotechnics and chemistry enthusiasts form a distinct group of HME producers. Modern technologies provide these individuals with unlimited access to diverse sources, such as scientific papers, books, manuals, patents, government reports, web tutorials, and discussion boards, contributing to the growing diversity and complexity of HMEs and IEDs.

This reality necessitates that law enforcement and other agencies devise new operating strategies, including in situ hazard identification, evidence collection and analysis methods, and monitoring suspicious activities on the web^17–26^. Simultaneously, legislators seek to restrict access to precursor components of HMEs (e.g., EU regulation no. 32013R0098). In addition to well-known and easily-obtainable explosive materials like triacetone triperoxide (TATP), hexamethylene triperoxide diamine (HMTD), lead or ammonium picrates, mercury fulminate or lead azide, security services occasionally discover other explosive compounds or mixtures (e.g., ethylene glycol dinitrate). While tracking orders for specific hazardous materials and confirming the presence of particular precursors in illegal laboratories (e.g., acetone, hydrogen peroxide, and acids for TATP) is possible for typical materials, the production or occurrence of other HMEs may go unidentified until they are criminally used.

Hydrogen peroxide is a vital chemical used in various areas of industries, medicine, household, hobbies, and more. It also serves as a crucial precursor of peroxide-based HMEs like TATP or HMTD^27,28^. Consequently, many countries, including the EU, impose regulations restricting private access to hydrogen peroxide. Solutions containing more than 12% w/w require special permission, and solutions exceeding 35% w/w are unavailable for consumers (regulation no. 32019R1148). Unfortunately, it is relatively easy to concentrate diluted solutions through distillation, freezing, or solvent extraction, making concentrated hydrogen peroxide available for illegal uses^29^. The purchase or confirmation of the presence of other HME’s precursors during searches, such as acetone, urotropine, and acids, in conjunction with concentrated hydrogen peroxide solutions, serves as an emergency signal. This circumstance is commonly known to officers and investigators.

However, concentrated hydrogen peroxide (50–60% w/w) forms a highly explosive and potent mixture with common powdered kitchen materials used as fuels, such as coffee, tea, flour, or spices. These materials may be considered as analogues of well-known, powerful liquid or gel explosives containing H_2_O_2_ and alcohols (e.g., glycerol, glycol, methanol)^30,31^. Such mixtures are known as HPOM (hydrogen peroxide-organic matter) systems. Data on grocery-based HPOMs are very sparse since only a few have been characterized. All of them have been classified as high-explosive materials. Their detonation velocities vary from 4700 to 6200 m/s, which is higher than that reported for ethanol-H_2_O_2_ mixtures (2250 m/s) and similar to the values for nitromethane-H_2_O_2_ explosives (6200 m/s). For comparison, the detonation velocity of TNT is approximately 6800 m/s. The explosion energy for grocery-based HPOMs, expressed as TNT equivalent, varies from 140 to 180%^32^. They function as secondary explosives, requiring primer material stimulation, but are highly dangerous. Identification of such a mixture is possible using standard peroxide tests^33–36^. However, the identification and detection of explosion residues and unexploded materials becomes more complicated, especially if the sample has been exposed to weather conditions or rinsed or diluted with water.

This study aims to identify markers of H_2_O_2_ oxidation in various powdered groceries and discuss their utility in detecting hydrogen peroxide-based explosives, even in materials collected as post-blast traces or samples discarded by the offender. The kinetics of their formation have been determined to assess the possibility of estimating the contact time of the oxidizer with fuel. The formation of these components was also examined in both stored dry and wet powders. For the experiments, grounded roasted coffee, sweet paprika, turmeric, and black tea from teabags were selected, as their mixtures with H_2_O_2_ exhibit the most potent explosive properties.

## Results

### IR spectroscopy

**Figure 1:**
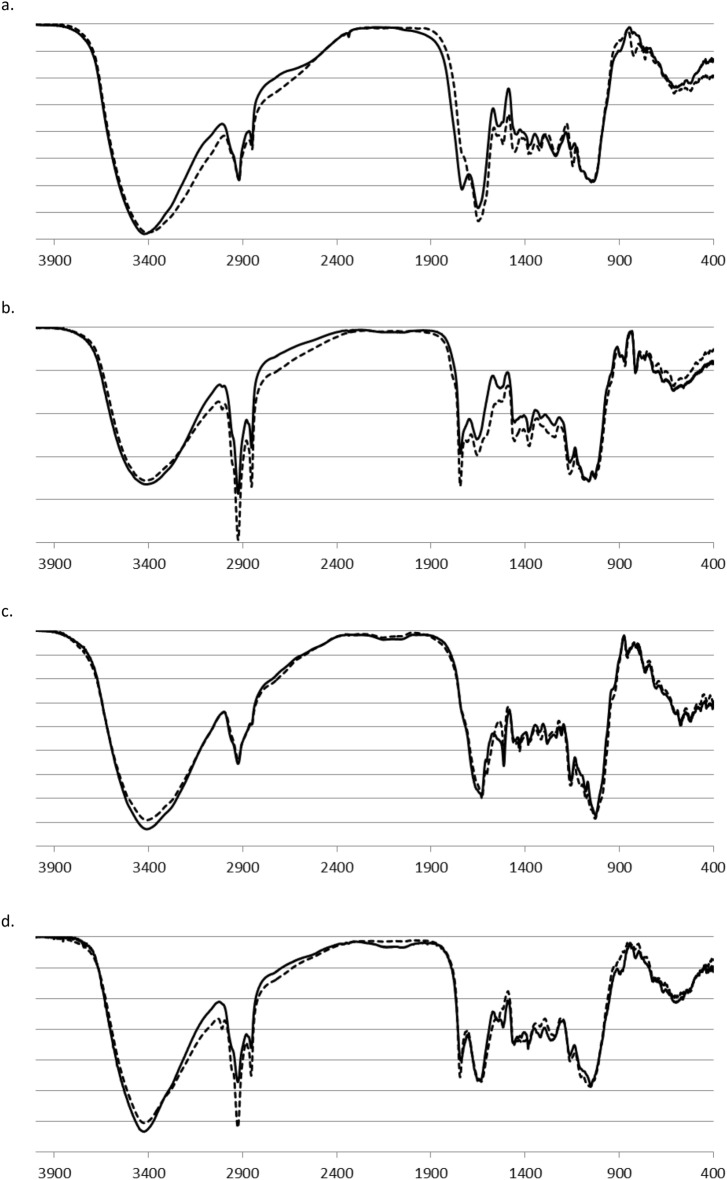
FT-IR spectra of samples before (‐‐‐‐‐) and after contact (60 min) with hydrogen peroxide (**——**): (**a**) black tea; (**b**) coffee; (**c**) turmeric; (**d**) sweet paprika.

The IR spectra of studied groceries before and after contact with hydrogen peroxide are presented in Fig. 1.

The spectra of untreated samples exhibit patterns typical for plant materials, with bands associated with polysaccharides, proteins, lipids, and lignins (including other polyphenols)^37^. Specific content values and major, characteristic IR bands for these components are summarized in Table 1^38–41^. Signals at 2850 and 2930 cm^−1^ correspond to stretching vibrations of the C-H bond of aliphatic groups, while bending vibrations of CH_3_ and CH_2_ groups show bands at approximately 1380 and 1430 cm^−1^. The absorption band at approximately 1640 cm^−1^ is related to water present in the samples.

**Table 1:** Major components of studied materials and characteristic IR bands.

	Black tea (%)	Coffee (%)	Turmeric (%)	Sweet paprika (%)	IR bands
Polysaccharides	22–27	55–65	60–70	50–55	3410–3450 cm^−1^ (O–H; st.) 1450 cm^−1^ (O–H, bend.) 1000–1250 cm^−1^ (O–H, bend.) 950–1050 cm^−1^ (C–O; st.)
Proteins	14–18	8–12	6–8	12–20	3200–3300 cm^−1^ (N–H; st.) 1620–1650 cm^−1^ (1° amide band) 1530–1550 cm^−1^ (2° amide band)
Lipids	1–3	11–16	2–7	7–16	3040 cm^−1^ (= C–H; st.) 1730–1750 cm^−1^ (C=O; st.) 1620–1650 cm^−1^ (C=C; st.) 1050–1250 cm^−1^ (C–O; st.)
Lignins & polyphenols	30–40	2–4	1–7	1–4	3410–3450 cm^−1^ (O–H; st.) 1710–1720 cm^−1^ (C=O; st.) 1610, 1515 cm^−1^ (phenyl ring deformation) 1000–1150 cm^−1^ (C–O; st.)

Changes in IR patterns after contact with hydrogen peroxide are minor and non-characteristic. The most noticeable differences occur for =C–H stretching vibrations related to unsaturated fatty acids (3040 cm^−1^). This peak is observed in lipids-rich materials, such as coffee and paprika, and decreases during reaction with H_2_O_2_. This variation results from C=C bond oxidation, leading to the formation of diols, epoxides, or chain breaking.

Other changes are less prominent. Some modifications may be observed in the 1480–1550 cm^−1^ region; however, their origin is challenging to explain as these wavenumber values are typical for various functional groups. Most likely, the oxidation of amines, such as amino acids or aminosaccharides, results in the formation of C-nitro and C-nitroso compounds, specifically the formation of the N=O bond. Therefore, bands in the discussed region represent stretching vibrations of the N=O group.

As shown, simple IR analysis of the examined material does not allow for the recognition of grocery samples that were components of powdered groceries-H_2_O_2_ explosive mixtures. Therefore, the most widely used methods for in situ risk assessment, such as Raman or FT-IR spectroscopy with portable infrared analyzers, are unsuitable for identifying these HMEs. However, some unspecific changes in relative band intensities and shapes offer a chance to address this issue after recording a considerable set of groceries-H_2_O_2_ HME samples and conducting spectra analysis using machine-learning methods.

### Gas chromatography

### Black tea

**Figure 2:**
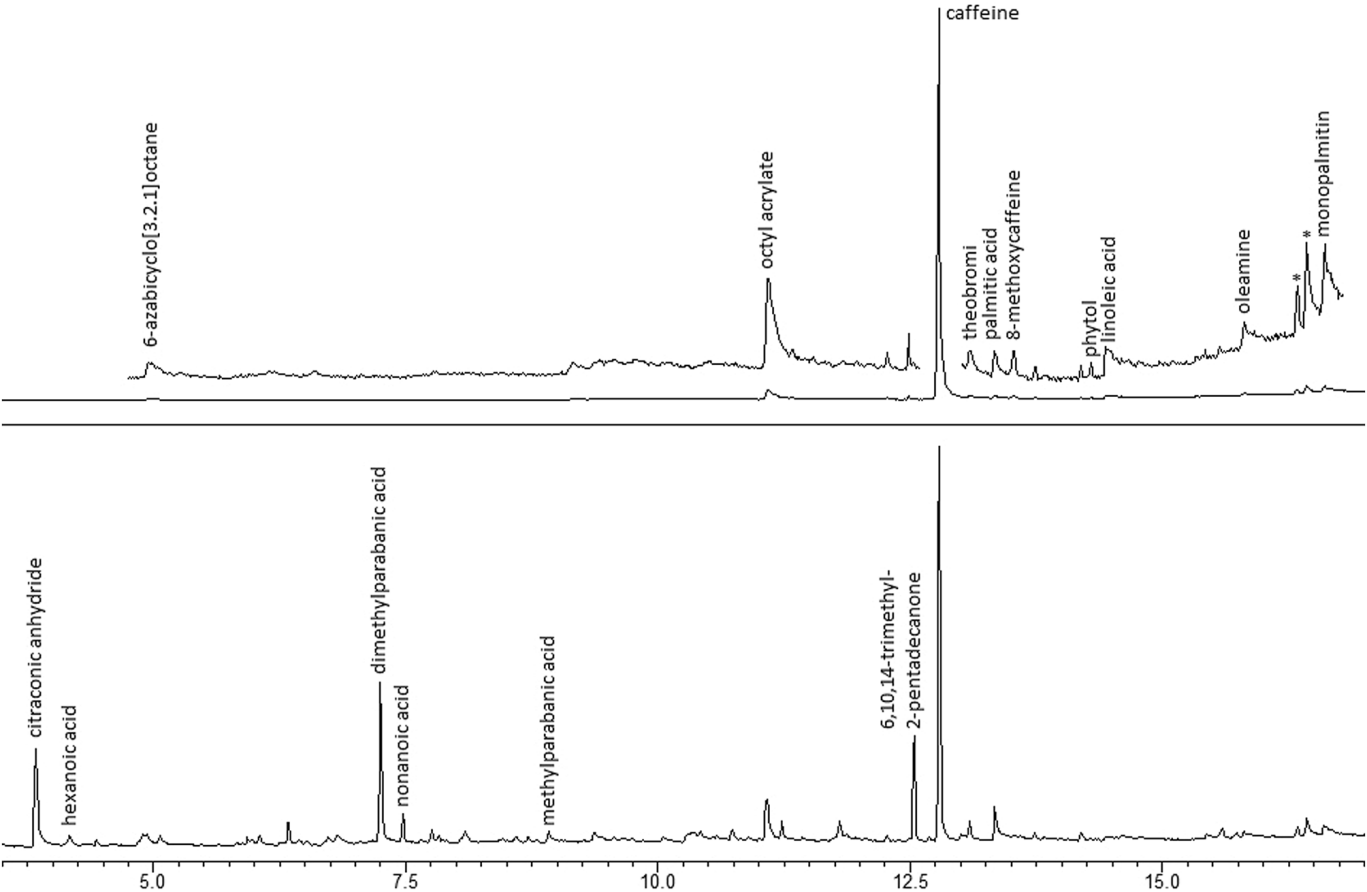
Total Ion Chromatograms (TIC) GC–MS of black tea (top) and H_2_O_2_-treated black tea (bottom); *—silicon-containing artifacts from column phase.

The gas chromatograms of methanolic extracts from black tea^42^ before and after treatment with hydrogen peroxide are presented in Fig. 2. The major component (refer to Tables 2 and 3) of the black tea extract is caffeine (CA), with traces of theobromine, palmitic acid, oleic acid, linolenic acid, phytol, and mono-palmitin (1- or 2-isomer). Additionally, impurities from the product container materials and/or those introduced during tea production were identified, including 6-azabicyclo[3.2.1]octane, oleamide, and octyl acrylate (or 2-ethylhexyl acrylate). It is probable that 8-methoxycaffeine detected forms spontaneously in the methanolic extract.

**Table 2:** Components detected in black tea extracts.

			
Caffeine	Theobromine	Methylparabanic acid	Dimethylparabanic acid
	
Phytol		6,10,14-trimethyl-2-pentadecanone	
	
Citraconic anhydride		*N*-acetylpiperidine

**Table 3:** Fatty acids and their cleavage products detected in studied samples.

	
Pentanoic acid	Hexanoic acid
	
Nonanoic acid	Myristic acid
	
Palmitic acid	Palmitoleic acid
	
Stearic acid	Oleic acid
	
Linoleic acid	Linolenic acid
	
1,1-dimethoxyhexane	1,1-dimethoxynonane
	
2-heptenal	2-octenal
	
2-decenal	4-decenal

2,4-decadienal	

In the extract from H_2_O_2_-treated black tea, several new compounds are present: methylparabanic acid, dimethylparabanic acid (DMPA), 6,10,14-trimethyl-2-pentadecanone, citraconic anhydride, *N*-acetylpiperidine, nonanoic acid and hexanoic acid.

Dimethyl- and methylparabanic acids are oxidation products of caffeine and theobromine, respectively^43–44^. Cleavage of the C=C bond leads to the formation of 6,10,14-trimethyl-2-pentadecanone (from phytol)^45^, nonanoic acid (from oleic acid and its esters), and hexanoic acid (from linoleic acid and its derivatives). Corresponding aldehydes in dimethyl acetal forms, i.e., 1,1-dimethoxyhexane and 1,1-dimethoxynonane, are present at low concentrations^46–50^. DMPA does not form in black tea during storage in contact with air, both in its dry and wet forms. The concentration of caffeine in black tea oxidized with H_2_O_2_ decreases sharply; after 60 min, its concentration is about one-tenth of the initial values. After 1 week of contact time, this compound becomes undetectable.

DMPA concentration increases monotonically in a series of samples with tea/H_2_O_2_ contact time ranging from 1 to 60 min but declines during one-week-long oxidation. Phytol undergoes rapid oxidation and is not detected in samples treated for 5 min. In contrast, its oxidation product, 6,10,14-trimethyl-2-pentadecanone is stable, and its concentration remains almost constant in all oxidized tea samples. The presence of dimethylparabanic acid is the best marker for tea treatment with hydrogen peroxide, as this product forms in relatively high amounts. However, this parameter may be used only for fresh samples. For old black tea material, which may have been treated with hydrogen peroxide for an extended period, the following properties indicated its HME character: the absence of caffeine, phytol, and unsaturated fatty acids, and the presence of 6,10,14-trimethyl-2-pentadecanone and short-chain aliphatic acids (C_6_ and C_9_).

The processes mentioned above were not observed for black tea samples stored in contact with air, in both dry and wet conditions. The only noticeable effect is the decrease of caffeine in tea washed with water. As a result, HPOM samples based on black tea can be easily distinguished from old and weathered tea residues.

**Figure 3:**
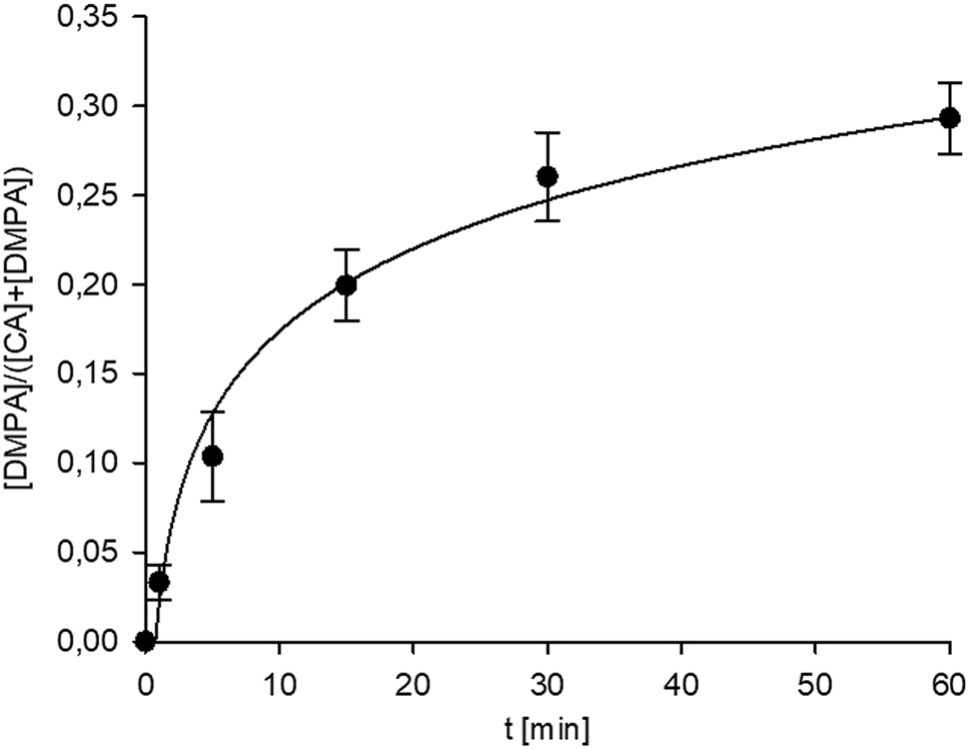
The time dependence of [DMPA]/([CA] + [DMPA]) ratio in black tea during H_2_O_2_ treatment.

The H_2_O_2_ treatment time can only be determined within a limited range through GC analyses. For the studied material, the [DMPA]/([DMPA] + [CA]) ratio shows a strong correlation with the reaction time (refer to Fig. 3). Absolute concentration values of these substances are poor predictors, as both compounds dissolve quite well in water. Therefore, their concentration in solid particles used for analysis decreases if the sample is washed with water or rinsed by rain. However, estimating these losses of analytes is difficult. Since H_2_O_2_ is used in HMEs in amounts significantly exceeding CA, the reaction can be approximated by a pseudo-first-order kinetic model. Due to further DMPA decomposition, the [DMPA]/([DMPA] + [CA]) ratio serves as a reliable predictor of the oxidation time for samples treated for 2 days or less. Unfortunately, there is no GC–MS marker suitable for determining the age of older samples.

### Coffee

**Figure 4:**
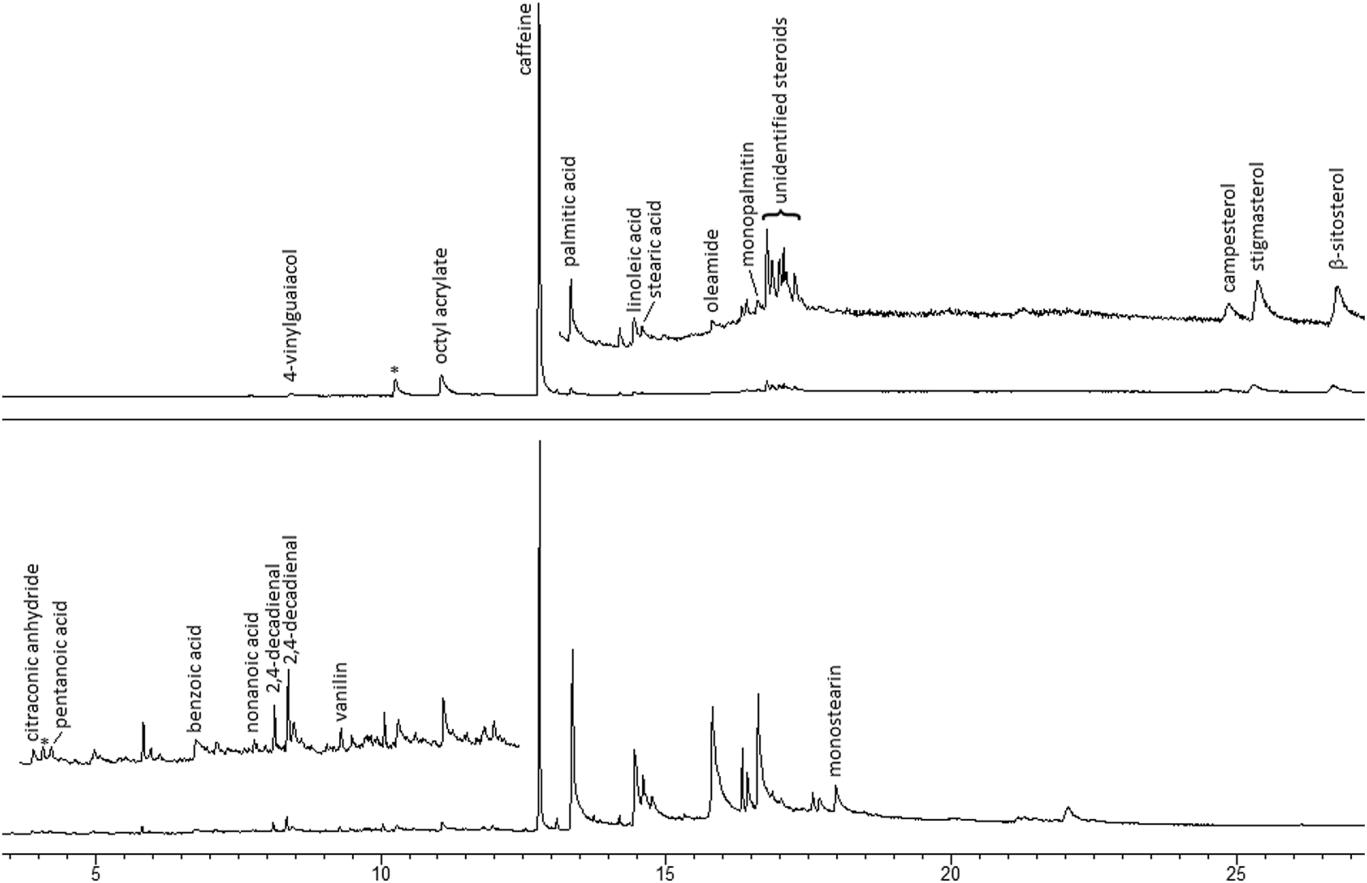
TIC GC–MS chromatograms of coffee (top) and H_2_O_2_ treated coffee (bottom); *—silicon-containing artifacts from column phase.

The TIC GC–MS chromatograms for untreated and H_2_O_2_-treated samples are presented in Fig. 4. Similar to black tea, the major component in the methanolic extract of coffee is caffeine (refer to Tables 3 and 4). Other minor compounds include fatty acids (palmitic, oleic, linoleic; free and as methyl esters formed spontaneously in methanol solution), monopalmitin, 4-vinylguaiacol, and 2,6-dimethylpyrazine. Traces of plant steroids, such as β-sitosterol, stigmasterol, and campesterol, were also detected^51,52^. Among artificial compounds, octyl acrylate and oleamide were identified.

**Table 4:** Components detected in coffee extracts.

	
4-vinylguaiacol	2,6-dimethylpyrazine
	
Benzoic acid	β-sitosterol
	
Stigmasterol	Campesterol

In opposition to black tea, the oxidation of caffeine in coffee comes more slowly. This is likely due to the high lipid content in powdered coffee. Firstly, this increases the material's hydrophobicity, inhibiting contact with hydrogen peroxide solution. Moreover, unsaturated fatty acid moieties act as peroxide scavengers, slowing down the oxidation of caffeine. As a result, the formation of dimethylparabanic acid (DMPA) is slow, and because this product undergoes subsequent reactions, DMPA does not accumulate in the sample and may be hard to detect. Small amounts of caffeine and DMPA are present even in coffee-based HPOM samples oxidized for over 1 week. Therefore, DMPA is a poor marker for HME mixtures based on the coffee/H_2_O_2_ system. Other compounds found in oxidized coffee powder include citraconic anhydride, benzoic acid, pentanoic acid, nonanoic acid, and 2,4-decadienal (two isomers).

The best markers indicating that coffee has been mixed with concentrated hydrogen peroxide are the decreasing ratios of linolenic to palmitic acid and caffeine to palmitic acid (note that this parameter may be affected by caffeine washing out by water). In our samples, the first parameter reduces from 2.12 to 1.15 and the second from 0.65 to 0.42 in a 60-min reaction time. However, these parameters do not permit establishing reaction time since they exhibit high variation and low repeatability.

Changes discussed above do not occur in ground coffee during its storage in contact with air, even in wet samples; therefore, they are specific for coffee-based HPOMs.

### Turmeric

**Figure 5:**
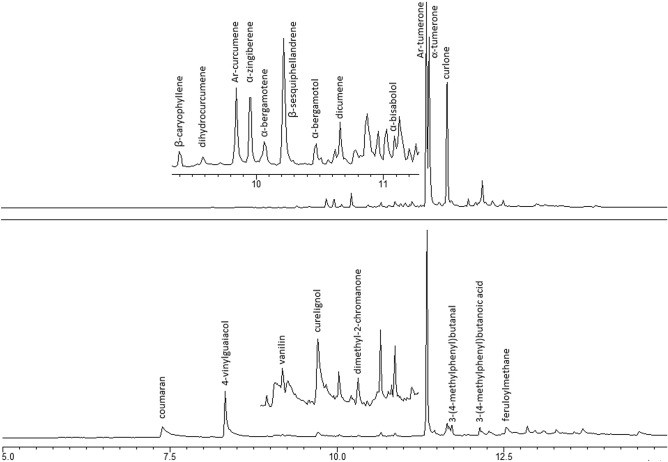
TIC GC–MS chromatograms of turmeric (top) and H_2_O_2_-treated turmeric (bottom).

Three major components (refer to Tables 3 and 5) of turmeric^53^ methanolic extract are ketones: Ar-turmerone, α-turmerone, and curlone. Among the minor substances are terpene hydrocarbons (β-caryophyllene, β-sesquiphellandrene, Ar-curcumene, dihydrocurcumene, α-zingiberene, α-bergamotene) and alcohols (bergamotol, α-bisabolol). An artificial component, dicumene, was also detected. However, several signals remain unidentified (refer to Fig. 5).

**Table 5:** Components detected in turmeric extracts.

		
Ar-turmerone	α-turmerone	Curlone
		
β-caryophyllene	β-sesquiphellandrene	Ar-curcumene
		
Dihydrocurcumene	α-zingiberene	α-bergamotene
		
α-bergamotol	α-bisabolol	Vanillin
		
cerulignol	4,7-dimethyl-2-chromanone	Coumaran
		
Feruloylmethane	3-(4-methylphenyl)butanoic acid	3-(4-methylphenyl)butanal

When treated with H_2_O_2_, the quickest decay is observed for α-turmerone; it is absent in samples after 1 min of oxidation. This instability is caused by the endocyclic cisoid 1,3-diene motif’s sensitivity to (per)oxidation^54,55^. Curlone is more stable; it is still detected in turmeric after 1 h of reaction with hydrogen peroxide. The aromatic Ar-turmerone is the most persistent and is present at small concentrations even after 1 week of reaction. In oxidized material, several compounds are identified, including 4-vinylguaiacol, vanillin, coumaran, cerulignol, dimethyl-2-chromanone (probably the 4,7-isomer), 3-(4-methylphenyl)butanal, feruloylmethane, 3-(4-methylphenyl)butanoic acid and monoglycerides.

4-Vinylguaiacol, vanillin, cerulignol, and feruloylmethane are products of curcuminoid degradation^56,57^, while 3-(4-methylphenyl)butanoic acid, 3-(4-methylphenyl)butanal and 4,7-dimethyl-2-chromanone form during the oxidation of Ar-turmerone and/or Ar-curcumene. The origin of coumaran is not clear.

The most exact markers for turmeric/H_2_O_2_-based HMEs are the absence of α-turmerone and only traces of curlone. For older samples, Ar-turmerone is also absent. On the other hand, the most characteristic markers of H_2_O_2_-oxidized samples, occurring in significant amounts, are coumaran and 4-vinylguaiacol.

The content of volatile components decreases slowly in turmeric during storage in open containers; however, even in one-year-old samples, sesquiterpenes are detected in high concentrations. α-Turmerone and curlone are air-sensitive, especially when the material is exposed to sunlight; therefore, their concentration decreases significantly over a few months, and these markers may not be present in weathered turmeric powder. Ar-turmerone is stable and does not decline in material stored in contact with air and/or water; therefore, its absence is a good marker for turmeric-based HPOM explosives. Also, above mentioned oxidation products (e.g., coumaran, 4-vinylguaiacol) do not form during turmeric storage, so their presence in the analyzed samples clearly indicates contact with H_2_O_2_.

**Figure 6:**
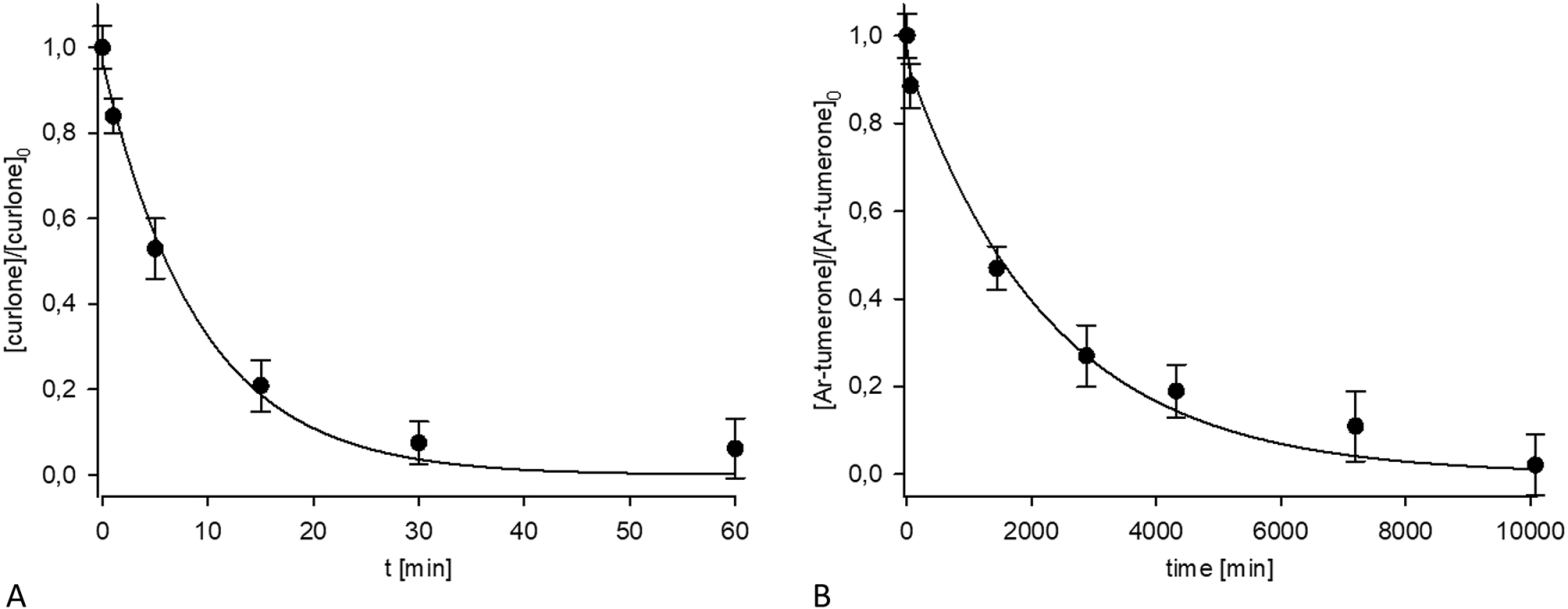
Time dependence of curlone (**A**) and Ar-turmerone (**B**) in turmeric during H_2_O_2_ treatment.

Decreasing Ar-turmerone and curlone concentrations may be used to estimate the contact time of the turmeric and hydrogen peroxide (refer to Fig. 6). These parameters differ in their application range. Ar-turmerone decays slowly, and its concentration may be reliably used for 1 day or older material, while curlone decreases within a few hours. There is no good marker for estimating a sample’s age longer than a week.

### Sweet paprika

**Figure 7:**
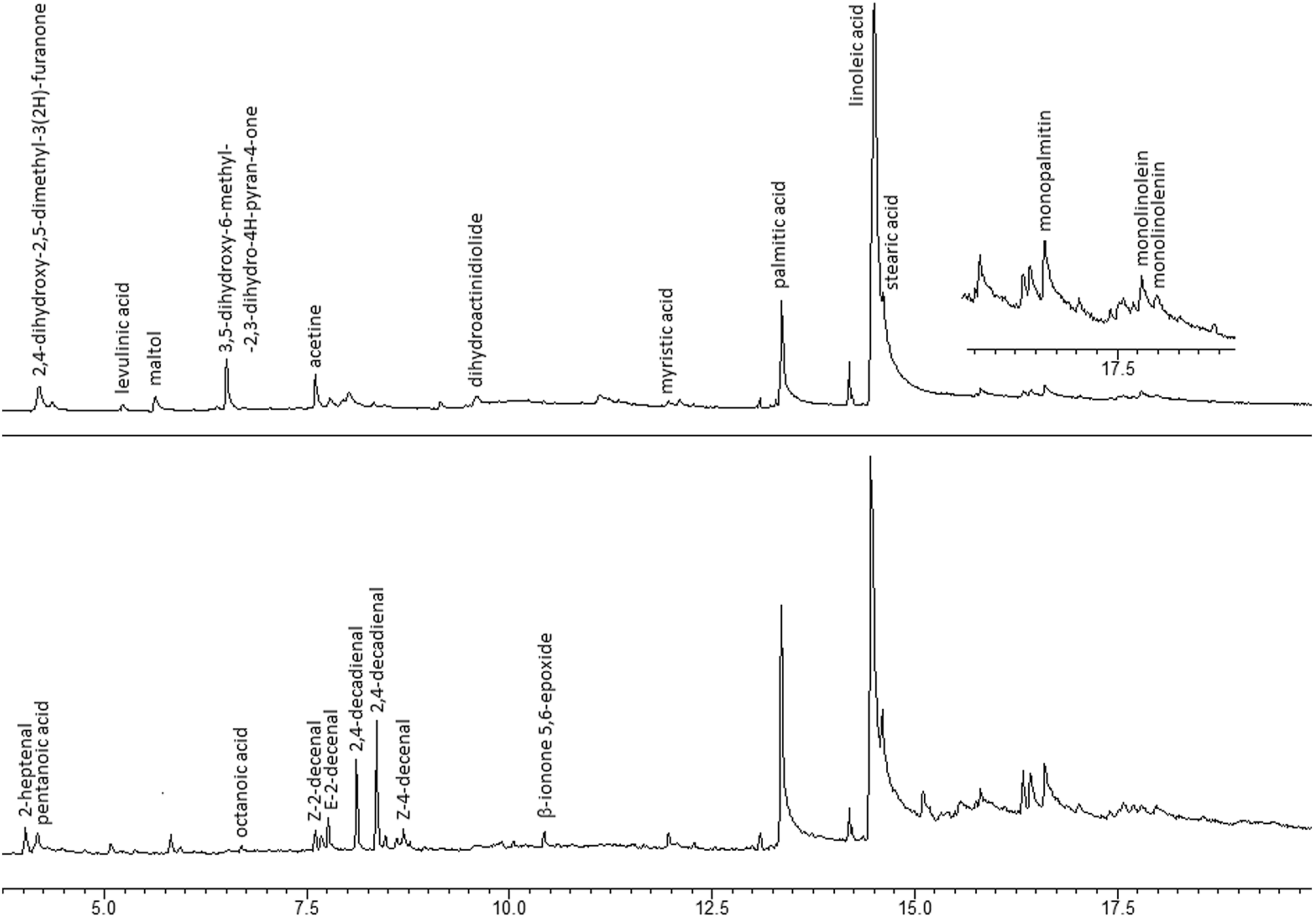
TIC GC–MS chromatograms of sweet paprika (top) and H_2_O_2_-treated sweet paprika (bottom).

The gas chromatogram of methanolic sweet paprika^58^ extract is relatively scanty (refer to Fig. 7). The major components (see Tables 3 and 6) are free fatty acids, including myristic, palmitic, palmitoleic, linoleic, and stearic acids. As trace ingredients, one may find monoglycerides (monopalmitin, monolinolenin, and monolinolein), 2,4-dihydroxy-2,5-dimethyl-3(2*H*)-furan-3-one, 3,5-dihydroxy-6-methyl-2,3-dihydro-4*H*-pyran-4-one, levulinic acid, maltol, 1-monoacetin, and dihydroactinidiolide. After contact with H_2_O_2,_ several new components form, including 2-heptenal, 2-octenal, 2-decenal (two isomers), 4-decenal, 2,4-decadienal (two isomers), pentanoic acid, hexanoic acid and nonanoic acid^49^. The formation of these molecules is related to the cleavage of unsaturated fatty acid moieties. Additionally, traces of epoxylated terpenoid—β-ionone 5,6-epoxide were found in some samples.

**Table 6:** Components detected in paprika extracts.

		
2,4-dihydroxy-2,5-dimethyl-3(2*H*)-furan-3-one	3,5-dihydroxy-6-methyl-2,3-dihydro-4*H*-pyran-4-one	Maltol
		
Levulinic acid	1-monoacetin	Dihydroactinidiolide

The most characteristic feature of H_2_O_2_-treated sweet paprika powder is the decreasing ratio of unsaturated fatty acids to total fatty acid content (from 0.89 ± 0.02 to 0.72 ± 0.05 in studied samples after 60 min of reaction). Another characteristic is the presence of unsaturated aldehydes, e.g., 2-heptenal; however, this parameter is less stable due to the volatility of these compounds. Unfortunately, the abovementioned parameters are unsuitable for estimating sample age or reaction time due to low repeatability.

The ratio of unsaturated fatty acids to total fatty acids is a stable parameter in sweet paprika samples stored for a long time in contact with air or moisture. The exposure of the material to sunlight may result in a decrease in linoleic acid content; however, other fatty acids detectable in extracts remain stable under such condition.

## Discussion

Dangerous and potent HMEs produced from powdered food products pose a challenging task for forensic scientists. However, GC–MS analyses allow for the detection of contact between grocery materials and hydrogen peroxide. The changes are particularly noticeable for some of the studied components (turmeric, black tea), while for others (coffee, sweet paprika), the changes are primarily quantitative. Changes detected in the former materials show clear time-dependency, enabling the determination of when the HME was prepared. However, among the studied materials, a suitable marker for age determination of older traces (approximately 1 week old) was identified only for turmeric. In general, studies of such mixtures should focus on the following stages:

- Protection of the sample, washing out hydrogen peroxide, extraction, and GC analysis (preparation of the extract should be made as soon as possible);
- Identification of oxidation markers—if present, HME production is highly probable;
- Quantitative comparison of oxidation markers concentration with those in original, untreated material;
- Reconstruction of the studied mixture and determination of the time dependence of HME markers’ concentration for the estimation of the original sample age.

The current preliminary results indicate the usefulness of the GC–MS technique for studying HMEs based on groceries and hydrogen peroxide mixtures (HPOM). Methanolic extracts obtained from HPOM samples remain stable over time, except for the formation of methyl esters from fatty acids and acetals from aldehydes. These processes need to be considered during the results analysis. The discussed changes (formation of oxidation products and the decrease in the concentration of some components of the original groceries) also occur at low or elevated temperatures (e.g., during winter or summer heat); therefore, HPOMs prepared in real, non-laboratory conditions can be identified. However, the kinetics of the oxidation processes are influenced by temperature, necessitating reconstruction at the actual temperature in all cases. Further areas of study include the analysis of HMEs containing other powdered food products (e.g., black pepper, flour) and the application of different analytical techniques, especially HPLC and SPME-GC chromatography.

## Methods

Caution! Concentrated hydrogen peroxide is corrosive to the skin. The mixtures described below warm up spontaneously and may ignite if prepared in larger amounts. Combinations of hydrogen peroxide with powdered groceries can violently explode when stimulated with a primary explosive!

### Materials

Hydrogen peroxide pure (Warchem, PL), with a nominal concentration 60% w/w and an actual concentration 56 ± 1% w/w, was used without purification; the concentration was determined by the standard iodometric procedure.

Phosphorus pentoxide (anhydrous, ReagentPlus^®^), methanol (Chromasolv^®^), *n*-dodecane (ReagentPlus^®^), and potassium bromide (Uvasol^®^) were obtained from Merck Life Science Sp.z.o.o. (PL).

Peroxides were detected in an aqueous solution using the peroxide strip test XploSens PS™ (Xplosafe LLC, USA).

Ground turmeric and sweet paprika (Kamis brand, McCormick Polska S.A., PL), ground coffee (Prima brand, Jacobs Douwe Egberts PL sp z.o.o., PL), and black tea from teabags (Minutka brand, Mokate S.A., PL) were used as obtained.

### General procedure

1 g of powdered grocery was mixed with 5 mL of hydrogen peroxide (56%). The samples were stored at room temperature (20 ± 1 °C). The reaction was quenched with water (20 mL), and the mixture was then centrifuged (4 000 rpm, 5 min). The solid fraction was washed several times with distilled water until a negative test for peroxides was obtained in the supernatant. The filtered-off solid material was dried in a desiccator over P_2_O_5_ for 2 h under vacuum. Oxidation was quenched after 1, 5, 15, 30, and 60 min. An additional sample of material that remained in contact with hydrogen peroxide for over 1 week was also prepared.

### FT-IR analyses

Transmission FT-IR spectra (4000–400 cm^−1^) were recorded using Bruker IFS 66v/S spectrometer. Samples were prepared as KBr pellets (1.5 mg of sample in 200 mg of KBr), and 256 scans were acquired.

### GC–MS analyses

25 mg of solid sample was suspended in 1 mL of methanol, and 5 µL of *n*-dodecane (internal standard) was added. The mixture was then sonicated in an ultrasonic bath for over 10 min at room temperature. Subsequently, the suspension was filtered through a syringe filter (pore size 0.45 µm) and analyzed using a Varian 4000GC/MS gas chromatograph equipped with a VF-5 ms column (30 m × 0.25 mm, *d*_*f*_ 0.25 µm; Agilent Technologies). The analysis parameters were as follows: carrier gas—helium, gas flow—1 ml/min, injector temperature—220 °C, column oven program—40 °C (isothermal, 3 min.), linear ramp 15 °C/min to 280 °C, 280 °C (isothermal, 10 min). Signals were identified using the NIST MS Search mass spectra library and literature data. Intensities are reported relative to the signal of internal standard (int. = 1.0).

## Data Availability

All the data are available on request from the corresponding author.
